# Iron Reduces the Trafficking of Fatty Acids from Human Immortalised Brain Microvascular Endothelial Cells Through Modulation of Fatty Acid Transport Protein 1 (FATP1/SLC27A1)

**DOI:** 10.1007/s11095-024-03743-w

**Published:** 2024-07-24

**Authors:** Showmika T. Supti, Liam M. Koehn, Stephanie A. Newman, Yijun Pan, Joseph A. Nicolazzo

**Affiliations:** 1 Drug Delivery, Disposition and Dynamics, Monash Institute of Pharmaceutical Sciences, Monash University, Parkville, VIC Australia; 2 Florey Institute of Neuroscience and Mental Health, University of Melbourne, Melbourne, VIC Australia

**Keywords:** blood-brain barrier, docosahexaenoic acid, fatty acid binding protein 5, fatty acid transport protein 1, iron

## Abstract

**Purpose:**

Alzheimer’s disease (AD) is associated with brain accumulation of amyloid-beta (Aβ) and neurofibrillary tangle formation, in addition to reduced brain docosahexaenoic acid (DHA) and increased brain iron levels. DHA requires access across the blood–brain barrier (BBB) to enter the brain, and iron has been shown to affect the expression and function of a number of BBB transporters. Therefore, this study aimed to assess the effect of iron on the expression and function of fatty acid binding protein 5 (FABP5) and fatty acid transport protein 1 (FATP1), both which mediate brain endothelial cell trafficking of DHA.

**Methods:**

The mRNA and protein levels of FABP5 and FATP1 in human cerebral microvascular endothelial (hCMEC/D3) cells was assessed by RT-qPCR and Western blot, respectively following ferric ammonium citrate (FAC) treatment (up to 750 µM, 72 h). The function of FABP5 and FATP1 was assessed via uptake and efflux of radiolabelled ^3^H-oleic acid and ^14^C-DHA.

**Results:**

FAC (500 µM, 72 h) had no impact on the expression of FABP5 at the protein and mRNA level in hCMEC/D3 cells, which was associated with a lack of effect on the uptake of ^14^C-DHA. FAC led to a 19.7% reduction in FATP1 protein abundance in hCMEC/D3 cells with no impact on mRNA levels, and this was associated with up to a 32.6% reduction in efflux of ^14^C-DHA.

**Conclusions:**

These studies demonstrate a role of iron in down-regulating FATP1 protein abundance and function at the BBB, which may have implications on fatty acid access to the brain.

**Graphical Abstract:**

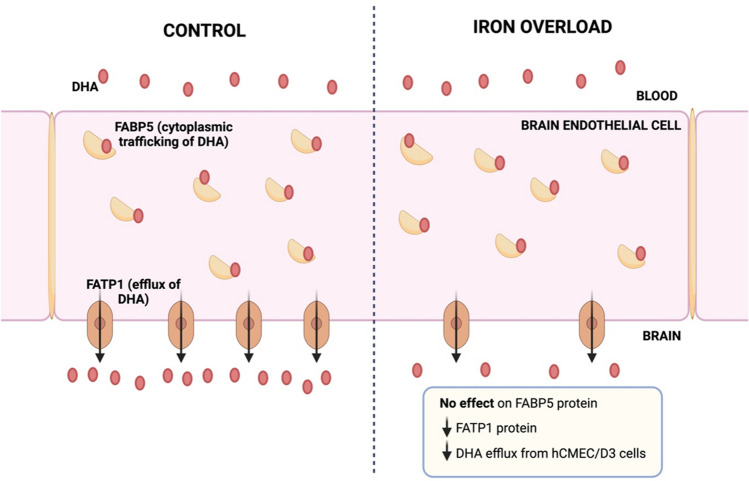

## Introduction

Alzheimer’s disease (AD) is a multifactorial neurodegenerative disease characterised by the brain accumulation of amyloid-β (Aβ), intraneuronal neurofibrillary tangles primarily composed of hyperphosphorylated tau, brain atrophy and neuroinflammation. In addition to these hallmark features, it has also been observed that docosahexaenoic acid (DHA) levels are reduced in AD brains [1–6]. DHA is a polyunsaturated fatty acid (PUFA) essential for the growth, development, and maintenance of normal brain function. DHA causes a reduction in Aβ load, phosphorylation of tau, and neuroinflammation in various AD mouse models [7]. There is a limited amount of DHA synthesised *de novo*, hence the brain primarily relies on plasma-derived DHA transported across the blood–brain barrier (BBB) to maintain the required DHA concentrations in the brain [8–10]. Given that BBB transport of DHA is important to maintain brain DHA levels, it is critical to understand how disease-related processes affect DHA trafficking across the BBB.

The BBB, a specialised structure composed of cerebral microvascular endothelial cells, is one of the barriers of the central nervous system (CNS) [11, 12]. The tight junctions between adjacent endothelial cells ensure that the majority of molecular trafficking across the BBB occurs via the transcellular pathway. The mechanisms driving the transport of DHA into the brain have been proposed to involve both passive diffusion and carrier mediated active transport. There are several classes of transport proteins that maybe responsible for protein-mediated fatty acid transport. Recent advances indicate that fatty acid binding protein 5 (FABP5) and fatty acid transport protein 1 (FATP1) which are highly expressed in the human brain microvessel endothelial cells (HBMEC) [13], are key contributors to brain endothelial cell trafficking, and therefore, brain access of DHA [14].

FABPs are chaperone proteins in the cytoplasm that have been implicated in the brain endothelial cell uptake and cytoplasmic trafficking of fatty acids [15]. An *in vitro* study showed that human FABP5 (hFABP5) expressed and purified from *E. coli* had high binding affinity to DHA [16]. This study further reported that genetic silencing of FABP5 with siRNA treatment caused a reduction in ^14^C-DHA uptake into human cerebral microvascular endothelial (hCMEC/D3) cells, an immortalised human brain endothelial cell line [16]. Importantly, DHA transport across the BBB was reported to be 14% lower in FABP5^−/−^ mice compared to wild type mice [17], resulting in lower endogenous DHA concentrations in the brain cortex and an impairment in cognitive function [17]. FATP1 is a membrane transporter suggested to facilitate the transport of fatty acids across the plasma membrane. Human embryonic kidney (HEK) 293 cells expressing FATP1 had significantly higher ^14^C-DHA uptake in comparison to mock HEK293 cells [18]. FATP1 has been shown to facilitate the transportation of ^14^C-oleate across HBMEC monolayers and specific siRNA knockdown of FATP1 reduced the ^14^C-oleate transport into both the luminal and abluminal medium [19], suggesting both an influx and efflux role of FATP1. Importantly, a recent study has shown that FATP1 genetic silencing led to a 47% reduction in the efflux of DHA-d5 from hCMEC/D3 cells [20]. As it has been shown that FATP1 is mainly expressed at the abluminal membrane of brain endothelium [18, 20], this membrane transporter is therefore suggested to efflux brain endothelial DHA into the brain parenchyma. Therefore, FABP5 is critical for the brain endothelial cell uptake and trafficking of DHA across the endothelial cell with FATP1 likely to be involved in the trafficking from the brain endothelial cell into the brain parenchyma.

The exact mechanisms that regulate the expression and function of FABP5 and FATP1, and whether these are modified in AD, are relatively unknown. However, studies conducted in various cell types suggest that FABP5 may be regulated via activation of peroxisome proliferator-activated receptors (PPARs). PPARγ agonists such as rosiglitazone, pioglitazone and troglitazone have been reported to increase the abundance of FABP5 protein in hCMEC/D3 cells, with pioglitazone increasing the levels and function of FABP5 at the BBB in mice [21]. On the other hand, DHA has also been shown to cause an upregulation of FATP1 and FATP4 in hCMEC/D3 cells following a 24-h treatment [22]. Some recent data suggest that amyloid beta_25-35_ down-regulates FATP1 and impairs DHA transportation in hCMEC/D3 cells [20]. However, the mechanism underlying how DHA and amyloid beta_25-35_ regulates FATP1 is unknown. Moreover, insulin treatment has been attributed to facilitate ^14^C-DHA transportation via increased translocation of FATP1 to the plasma membrane in hCMEC/D3 cells [18].

Neurological disease states, such as AD, exhibit well defined elements of metal ion dyshomeostasis. Evidence in the literature shows that there is a significant elevation of iron levels in the cortical and hippocampal regions (the main brain areas affected in AD) during preclinical stages of AD [23, 24]. Moreover, iron overload has been linked to the rate of cognitive decline [25], and it is also proposed that iron deposition in the hippocampal region could be used as a predictor for the rate of cognitive decline associated with Aβ [26]. There are many studies reporting on the effects of iron on the pathology of AD [25, 26], and now a growing appreciation of the effects of iron on the BBB, which is also affected in AD [27]. For example, the function of tight junction protein occludin at the BBB has been shown to be reduced due to iron overload in the rat BBB [28]. To this end, our laboratory has recently assessed the impact of iron on other key transporters at the BBB. In particular, Newman *et al*. demonstrated that ferric ammonium citrate (FAC) downregulated P-glycoprotein (P-gp) mRNA expression and a subsequent protein abundance by 36% in hCMEC/D3 cells [29], an effect which was associated with iron-mediated reactive oxygen species (ROS). Moreover, FAC treatment also reduced breast cancer resistance protein (BCRP) mRNA expression in hCMEC/D3 cells, an effect mediated through the ability of FAC to increase the phosphorylation of ERK1/2 [30]. Iron dextran treatment has also been shown to increase mRNA expression of FABP1 in hepatocytes of eight-week-old male Fischer rats, however the study was only limited to analysis of the gene profile and did not investigate changes to protein levels [31]. Therefore, this current study aimed to determine the effect of iron, in the form of FAC, on the expression and function of FABP5 and FATP1 and on DHA transport in hCMEC/D3 cells, as a model of the human BBB.

## Materials and Methods

### Materials

The hCMEC/D3 cells were kindly provided by Dr. Pierre-Olivier Couraud (INSERM, Paris, France). Dimethyl sulfoxide (DMSO), beta-mercaptoethanol (β-ME), N-2-hydroxyethylpiperazine-N-2-ethane sulfonic acid (HEPES), penicillin/streptomycin, absolute ethanol, sodium dodecyl sulphate (SDS), thiazolyl blue tetrazolium bromide (MTT), dichlorofluorescin diacetate (DCFH-DA), Triton X-100, Tween 20, Dulbecco’s phosphate buffer saline (D-PBS), trypan blue, bovine serum albumin (BSA), FAC, N-Acetyl-L-cysteine (NAC) and sodium pyruvate (SP) was purchased from Sigma-Aldrich (St Louis, MO). Endothelial basal medium-2 (EBM2) media and EGM-2 SingleQuots growth factor kits were purchased from Lonza (Walkersville, MD). Trypsin EDTA was purchased from Life Technologies (Carlsbad, CA). Rat-tail collagen Type I and all plastic cell culture equipment were purchased from Corning (Corning, NY). Hank’s balanced salt solution (HBSS), Pierce bicinchoninic acid (BCA) protein assay kit, and Pierce IP lysis buffer were purchased from ThermoFisher Scientific (Rockport, IL). Complete mini protease inhibitor cocktail tablets were purchased from Roche (Mannheim, Germany). Amersham Protran 0.2 μm nitrocellulose membranes were purchased from GE Healthcare Life Sciences (Little Chalfont, UK). Precision Plus Protein Dual Xtra Standards, iTaq Universal Probes One-Step kit, Mini-Protein TGX Precast gels (4–20% acrylamide) and extra thick blot paper were purchased from Bio-Rad (Hercules, CA). Intercept (PBS) Blocking Buffer and Licor donkey anti-rabbit antibody (680 nm) were purchased from Millennium Science (Melbourne, Victoria, Australia). Rabbit polyclonal antibody to FABP5 (ab37267) and anti-β-actin antibody (ab179467) were obtained from Abcam (Cambridge, UK). IHC‑plus polyclonal rabbit anti‑human SLC27A1 / FATP antibody (IHC, WB) LS‑B16490 was purchased from LifeSpan BioSciences (Seattle, WA), Hs_SLC27A1_6 FlexiTube siRNA (GeneGlobe ID—SI04152603|S2) (1027418), AllStars Negative Control siRNA (1027280), HiPerFect Transfection Reagent (301705), QIAshredder columns and RNeasy Plus Mini kit were purchased from Qiagen (Hilden, Germany). Taqman gene assays for FABP5 (#Hs_02339439_g1, FAM), FATP1 (#Hs_01587911_m1, FAM), β-actin (#Hs_01060665_g1, FAM), and GAPDH (#Hs_0227258991_g1, FAM) were purchased from Applied Biosystems (Foster City, CA). Pioglitazone (AG-CR1-0067-M005) was obtained from AdipoGen Life Sciences (San Diego, CA). ^3^H-Oleic acid and ^14^C-docosahexaenoic acid were purchased from American Radiolabelled Chemicals (St. Louis, MO). Ultima Gold liquid scintillation cocktail was obtained from PerkinElmer Life Sciences (Waltham, MA).

### Culturing of hCMEC/D3 Cells

hCMEC/D3 cells were maintained in T75 flasks pre-coated with 0.1 mg/mL solution of rat-tail collagen. The cells were incubated at 37 ºC, with an air composition of 95% O_2_ and 5% CO_2_, and in EBM-2 media supplemented with vascular endothelial growth factor (0.025% v/v), insulin-like growth factor 1 (0.025% v/v), epidermal growth factor (0.025% v/v), basic fibroblast growth factor (0.1% v/v), hydrocortisone (0.01% v/v), ascorbic acid (0.01% v/v), fetal bovine serum (2.5% v/v), 10 mM HEPES (pH 7.4), gentamicin-amphotericin (0.01% v/v) and penicillin − streptomycin (1% v/v). For experiments, hCMEC/D3 cells between passages 28 to 35 were seeded at a density of 20,000 cells/cm^2^ into collagen-coated multi-well plates. Cells plated in 6-well plates were used for qPCR and western blot analysis, cells seeded in 24-well plates were used for uptake and efflux studies, and cells seeded in 48-well plates were used to assess ROS levels.

### Treatment of hCMEC/D3 Cells

Pioglitazone, a known PPARγ agonist, was used as a positive control treatment (25 µM, 72 h) because it is known to upregulate FABP5 in hCMEC/D3 cells [21]. To determine the effects of FAC on hCMEC/D3 cells, a 72 h treatment (500 µM) was used. To determine whether the effects of FAC on FATP1 were ROS-mediated, hCMEC/D3 cells were treated with FAC (500 µM) in the presence of NAC (10 mM) or SP (10 mM), on account of their established antioxidant effects *in vitro* and *in vivo* [28, 29, 32, 33].

To assess the involvement of FATP1 in the transportation of various fatty acids, an siRNA treatment was conducted to silence the expression of FATP1. For the treatment, 6 well plates were seeded and allowed to incubate for 4–5 h, before being treated with either 50 nM of scrambled RNA (control) or 50 nM of FATP1 specific siRNA, which was repeated at 24 h post seeding, to ensure that the treatment for this group mimicked the 72 h FAC treatment.

**Fig. 1:**
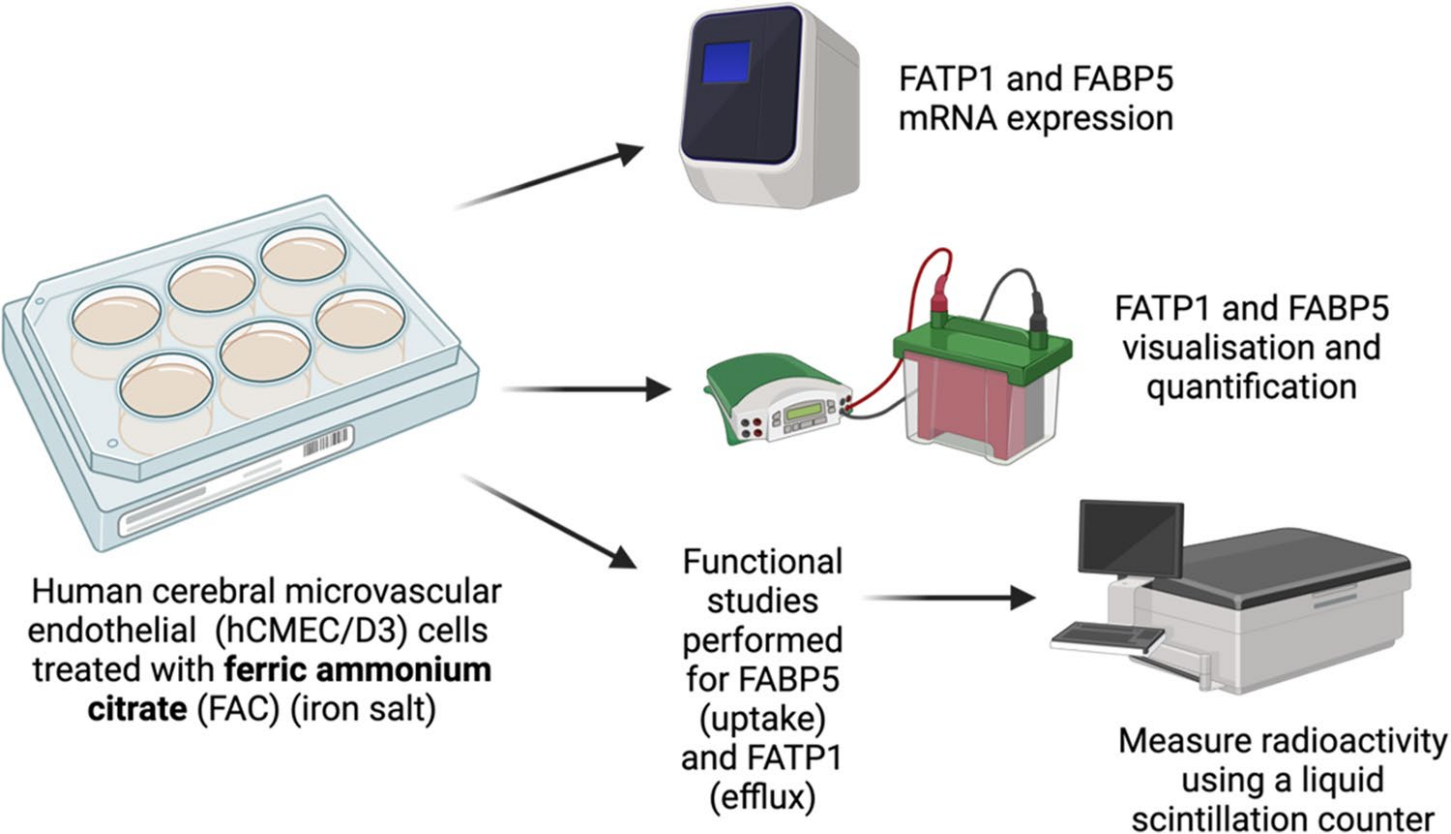
The schematic diagram illustrates the workflow for studying the expression and quantification of FATP1 and FABP5 using key steps that include mRNA expression analysis by RT-qPCR, protein visualisation and quantification by western blotting and functional studies (uptake and efflux studies) using radioactive ^3^H-OA and ^14^C-DHA

Following treatments, cells were then used for mRNA expression studies (using qPCR), protein abundance studies (using western blots) or to assess function of FABP5 and FATP1 (using uptake and efflux studies), as shown in Fig. 1.

### MTT Assay

At the end of the relevant treatment period, hCMEC/D3 cells were incubated with 150 μL of 0.45 mg/mL MTT reagent solution in blank EBM-2 media for 3 to 4 h to allow the cells to reduce the MTT. Next, the MTT reagent was carefully removed from the wells and then replaced with 150 μL of DMSO to dissolve the resulting purple formazan. After a further 30 min of incubation, the plates were agitated, and absorbance was read at 540 nm using a PerkinElmer multimode plate reader (Waltham, MA). Background absorbance (average reading of blank wells) was subtracted from all treatment wells before analysis. The absorbance readings of treatment wells were normalised against the average of the control wells and cell viability with each treatment expressed as a percentage to the control.

### Western Blot for Quantification of Protein Abundance of FABP5 and FATP1 in hCMEC/D3 Cells

At the end of the relevant treatment period, hCMEC/D3 cells were lysed using Pierce IP lysis buffer containing Roche Complete mini protease inhibitor cocktail. The Pierce BCA Protein assay kit was used according to the manufacturer’s protocol to determine total protein concentrations against BSA standards. The samples were analysed at 546 nm using a PerkinElmer multimode plate reader (Waltham, MA) to obtain the absorbance values.

The prepared lysates (10 µg) were mixed with loading (Laemmli) buffer (consisting of 20% (w/w) glycerol, 0.125 M Tris–HCl buffer, 10% (v/v) SDS, 0.5% (v/v) bromophenol blue and 25% (v/v) β-mercaptoethanol in milli-Q (MQ) water), in a ratio of 5:1 and incubated for 10 min at 70°C. The samples were then loaded into a Bio-Rad 4–20% acrylamide pre-cast gel. The Bio-Rad mini protean tetra cell (Hercules, CA) was used for the gel electrophoresis. The separation was completed at 200 V for approximately 50 min or until the bands reached the reference line on the gel. Once the separation was complete, the proteins from the gel were transferred to a 0.2 µm nitrocellulose membrane at 11 V for 42 min using a Bio-Rad semi-dry transblot (Hercules, CA). Upon completion of the transfer, the membrane was incubated in either Intercept (PBS) blocking buffer (for FABP5) or 5% non-fat milk (for FATP1) for 2 h, to prevent non-specific antibody binding. The membrane was then incubated overnight at 4°C under gentle agitation with primary antibodies specific to human FABP5 (1:500), human FATP1 (1:4000), and primary β-actin (1:10000) that were diluted using Intercept (PBS) blocking buffer. The membrane was then incubated with donkey anti-rabbit secondary antibody (1:30000), diluted using Intercept (PBS) blocking buffer, for 2 h at room temperature in a light-proof container. The membrane was then imaged using an Amersham Typhoon 5 imaging system (GE Healthcare, Little Chalfont, Buckinghamshire, United Kingdom) and analysed using Image J (National Institutes of Health, Bethesda, MA), with determination of the relative protein abundances assessed by densitometric analysis. The abundance of FABP5 or FATP1 protein was quantified relative to β-actin (housekeeping protein), and fold-changes in treated groups were calculated as a ratio to the control.

### RNA Isolation and RT-qPCR to Assess mRNA Expression of FABP5 and FATP1 in hCMEC/D3 Cells

After the cells were treated for the desired period, RNA isolation was performed as per the manufacturer’s protocol using the RNeasy Plus Mini kit. The concentration of the eluted RNA was then measured using a NanoDrop 1000 spectrophotometer (Thermo Fisher Scientific, Waltham, MA). RT-qPCR was conducted on a Bio-Rad C1000 thermocycler in a CFX96 system (Hercules, CA) to quantify FABP5 and FATP1 gene expression. As per the manufacturer’s protocol, each PCR reaction mix contained 12.5 μL of 2 × probe RT-PCR reaction mix, 0.5 μL of iScript reverse transcriptase, 6.3 μL of nuclease-free water, 0.695 μL of Taqman primer, and 100 ng of RNA (in 5 μL), which was added into each well of a 96 well Thermowell Gold PCR plate. The plate was carefully sealed using optical tape and vortexed. The plate was then centrifuged at 2000 *xg* for 5 min at 4°C. RT-qPCR was performed at 50°C for 10 min, 95°C for 5 min, and then 50 cycles of 95°C for 15 s and 60°C for 30 s. To determine relative gene expression between control and treated cells, the fold-change method (2^−ΔΔCt^) was employed, according to Equations (Eq) below. Expression was normalised to the geometric mean of the housekeeping genes, GAPDH and β-actin. Where the treatment caused an effect to the cycle threshold values (C_t_) of β-actin, only GAPDH was used to normalise the data.

1 $${\Delta C}_t=C_{(FABP5\;or\;FATP1)t}-(C_{t\left(\beta-actin\right)}+C_{t(GAPDH)})/2$$

2 $${\Delta \Delta C}_{t}={C}_{t (treated)}-{C}_{t (control)}$$

3 $$2^{-\Delta\Delta Ct}=fold-change\;of\;FABP5\;or\;FATP1\;normalised\;to\;\beta-actin\;and\;GAPDH$$

### Uptake and Efflux of ^3^H-OA and ^14^C-DHA in hCMEC/D3 Cells

In order to examine the effect of FAC and pioglitazone treatment on FABP5 function in hCMEC/D3 cells, cellular uptake studies were performed. Following treatment with pioglitazone (as a positive control) or FAC, the cells were rinsed with 100 μL of pre-warmed uptake buffer (5% (w/v) HEPES in HBSS, pH 7.4) before being supplemented with 200 μL of fresh pre-warmed uptake buffer containing 0.1 μCi of ^3^H-Oleic acid (^3^H-OA), a surrogate for DHA or 0.2 μCi of ^14^C-DHA, per well [34]. The plate was left on the Thermostar (BMG Labtech, Ortenberg, Germany) at 37°C, 200 rpm. The uptake studies were ceased after 1 min by removing ^3^H-OA or ^14^C-DHA from all wells to stop cellular uptake and rinsing the cells with ice-cold HBSS twice. The end point for the uptake was 1 min since it was previously shown that the uptake of ^3^H-OA into hCMEC/D3 was linear up until this time [34]. The cells were then incubated with 100 μL of lysis buffer for 30 min at temperatures between 2–8 ºC, following which, 60 μL of cell lysate was removed and all samples were thoroughly vortexed with 2 mL of scintillation fluid (Ultima Gold Cocktail), and radioactivity was determined using a PerkinElmer 2800TR liquid scintillation counter (Waltham, MA). Total cell protein counts were performed with a 20 μL aliquot of cell lysate using the Pierce BCA protein assay kit. The cellular uptake was calculated as described in Eq. 4.

4 $$\text{Cellular uptake }\left(\mathrm{mL}/\mathrm{mg}\right)= \frac{\frac{\text{Radiolabelled compound in lysate }(\mathrm{DPM})}{\text{protein count }(\mathrm{mg})}}{\text{Radiolabelled compound in uptake buffer }(\frac{\mathrm{DPM}}{\mathrm{mL}})}$$

Cellular efflux studies were also performed in hCMEC/D3 cells given the observations that FAC impacted on FATP1 abundance and the reports that FATP1 has been implicated in the efflux of fatty acids [20]. Following treatment with FAC and/or FATP1 siRNA, hCMEC/D3 cells were washed twice with 400 µL of pre-warmed efflux buffer, 5% (w/v) HEPES in HBSS (pH 7.4). The cells were then incubated in 200 µL of efflux buffer for 10 min at 37°C. At the completion of the incubation period, hCMEC/D3 cells were loaded with ^3^H-OA or ^14^C-DHA by exposing them to 200 µL of pre-warmed ^3^H-OA (0.1 µCi) or ^14^C-DHA (0.2 µCi) (prepared in efflux buffer with 1:1 molar ratio of fatty acid free BSA) for 30 min. The hCMEC/D3 cells were then washed thrice with 400 µL of pre-warmed efflux buffer, after which, the cells were then exposed to 200 µL of pre-warmed blank efflux buffer. The plate was left on the Thermostar at 37°C, 200 rpm for either 2, 5, 10, or 20 min, after which 100 µL of efflux buffer was collected. The cells were immediately washed with 400 µL of ice-cold HBSS containing 0.1% fatty acid free BSA four times and 100 µL of lysis buffer was added to each well to perform the protein count. To measure the amount of ^3^H-OA or ^14^C-DHA that had effluxed from the cells into the efflux buffer at each timepoint, a 100 µL sample of efflux buffer was vortexed with 2 mL of scintillation fluid (Ultima Gold Cocktail) and radioactivity was determined using a PerkinElmer 2800TR liquid scintillation counter. To determine the amount of ^3^H-OA or ^14^C-DHA remaining in the cells post efflux, 60 μL of cell lysate was thoroughly vortexed with 2 mL of scintillation fluid (Ultima Gold Cocktail), and radioactivity was determined using a PerkinElmer 2800TR liquid scintillation counter. Total cell protein counts were performed with a 20 μL aliquot of cell lysate using the Pierce BCA protein assay kit. The cellular efflux amount of ^3^H-OA and ^14^C-DHA and the amount of ^3^H-OA and ^14^C-DHA remaining in cells post efflux was calculated via Eq. 5 and Eq. 6, where uptake buffer refers to the solution to which the cells were originally exposed.

5 $$\text{Efflux amount }\left(\frac{\mathrm{mL}}{\mathrm{mg}}\right)= \frac{\frac{\text{Radiolabelled compound in efflux buffer }\left(\mathrm{DPM}\right)}{\text{Protein count }\left(\mathrm{mg}\right)} }{\text{Radiolabelled compound in uptake buffer }(\frac{\mathrm{DPM}}{\mathrm{mL}})}$$

6 $$\text{Amount remaining in cells }\left(\frac{\mathrm{mL}}{\mathrm{mg}}\right)= \frac{\frac{\text{Radiolabelled compound in lysate }\left(\mathrm{DPM}\right)}{\text{protein count }\left(\mathrm{mg}\right)}}{\text{Radiolabelled compound in uptake buffer }\left(\frac{\mathrm{DPM}}{\mathrm{mL}}\right)}$$

### Quantification of Intracellular ROS in hCMEC/D3 Cells

At the end of the treatment period, hCMEC/D3 cells were rinsed twice with ‘assay medium’ consisting of blank EBM-2 medium containing 10 mM HEPES (pH 7.4). Following this rinse, 250 μL of assay medium containing 20 μM DCFH-DA was then added to the vehicle and FAC treated wells and blank assay medium was added to the untreated wells (to correct for background cellular autofluorescence). The plate was then incubated for 30 min at 37°C. Following incubation, the wells were rinsed twice and 250 μL of assay medium was added to each well. The plate was then scanned immediately to measure fluorescence from underneath in 25 areas at 0.72 mm intervals (λex: 485 nm, λem: 535 nm) using the Perkin Elmer multimode plate reader (Waltham, MA). After reading the fluorescence, the cells were lysed and protein count from each well was performed using the BCA assay. ROS levels in each well were expressed as the ratio of corrected DCF fluorescence to the protein count per well in μg.

## Statistical analysis

All data were analysed using GraphPad Prism 10 (GraphPad Software Incorporated, La Jolla, CA) and are expressed as mean ± SD. When comparing between two treatment groups, a Student’s unpaired t-test was performed and when more than two treatment groups were compared, an analysis of variance (ANOVA) followed by a post-hoc test was performed. A post-hoc Dunnett’s test (comparing the mean of each treatment to the control mean) and a post-hoc Tukey’s test (comparing the mean of every treatment to all other treatments) were performed post one-way ANOVA. A post-hoc Šídák's test was performed between to compare means between control and treated cells at varied time points following a two-way ANOVA test. The test performed is stated in the figure legends, and p < 0.05 was considered statistically significant. The number of biological replicates in the *in vitro* studies for independent cell culture preparations are represented as ‘n’ in each figure legend. All data points were used for statistical analysis and no sample size calculation was performed.

## Results

### Determination of Tolerable Non-Toxic Concentrations of FAC in hCMEC/D3 Cells

**Fig. 2:**
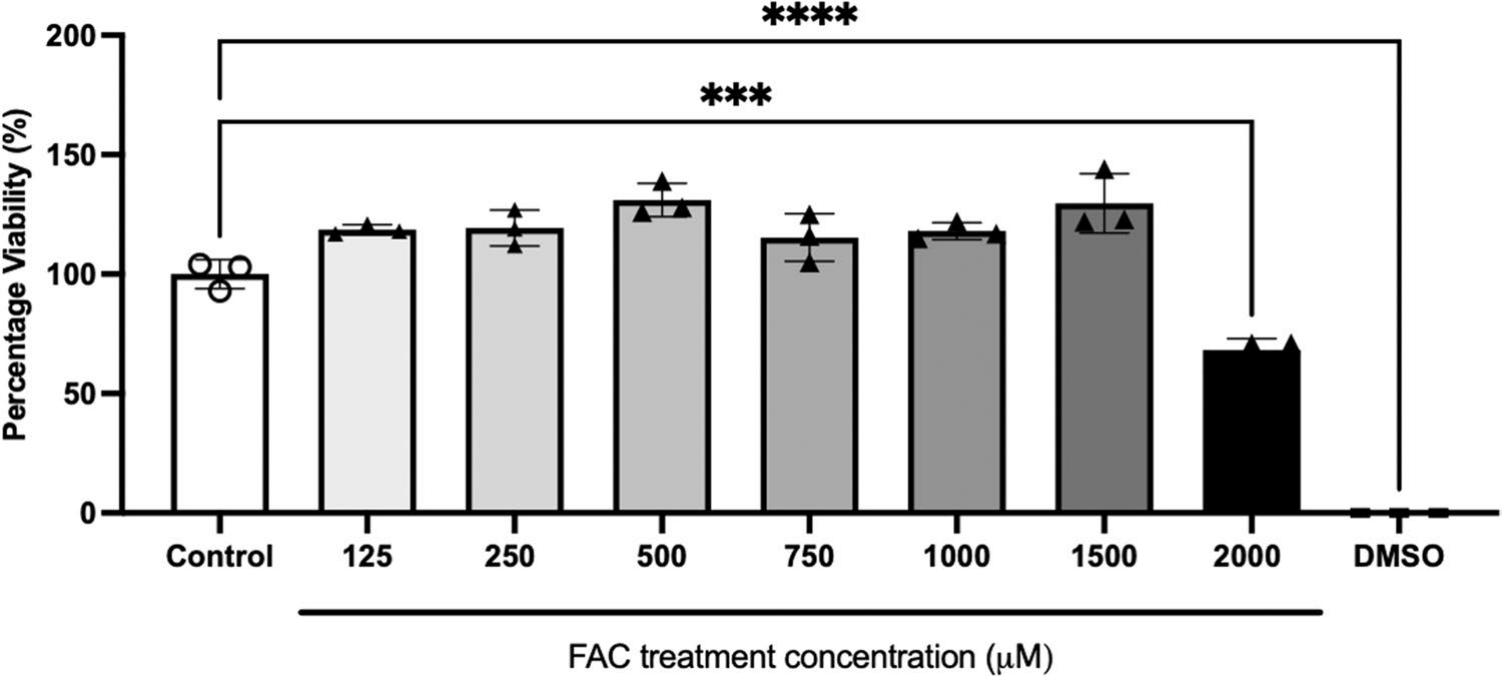
hCMEC/D3 cell viability assay when treated with vehicle, 125 to 2000 µM FAC and 10% (v/v) DMSO (as positive control) over 72 h. Data are presented as mean ± SD (*n* = 3, independent cell culture preparations). ***p < 0.001 and ****p < 0.0001, using a one-way ANOVA and a post-hoc Dunnett’s test where the mean of every treatment was compared to the control mean

The MTT assay was conducted on hCMEC/D3 that were exposed to FAC at concentrations ranging between 125 to 2000 µM for 72 h (Fig. 2). A concentration of 1500 µM was identified to be the highest tolerable concentration of FAC and concentrations lower than 1500 µM were then used for further experiments with a treatment period of up to 72 h.

### Effect of FAC and Pioglitazone on Abundance of FABP5 Protein in hCMEC/D3 Cells

**Fig. 3:**
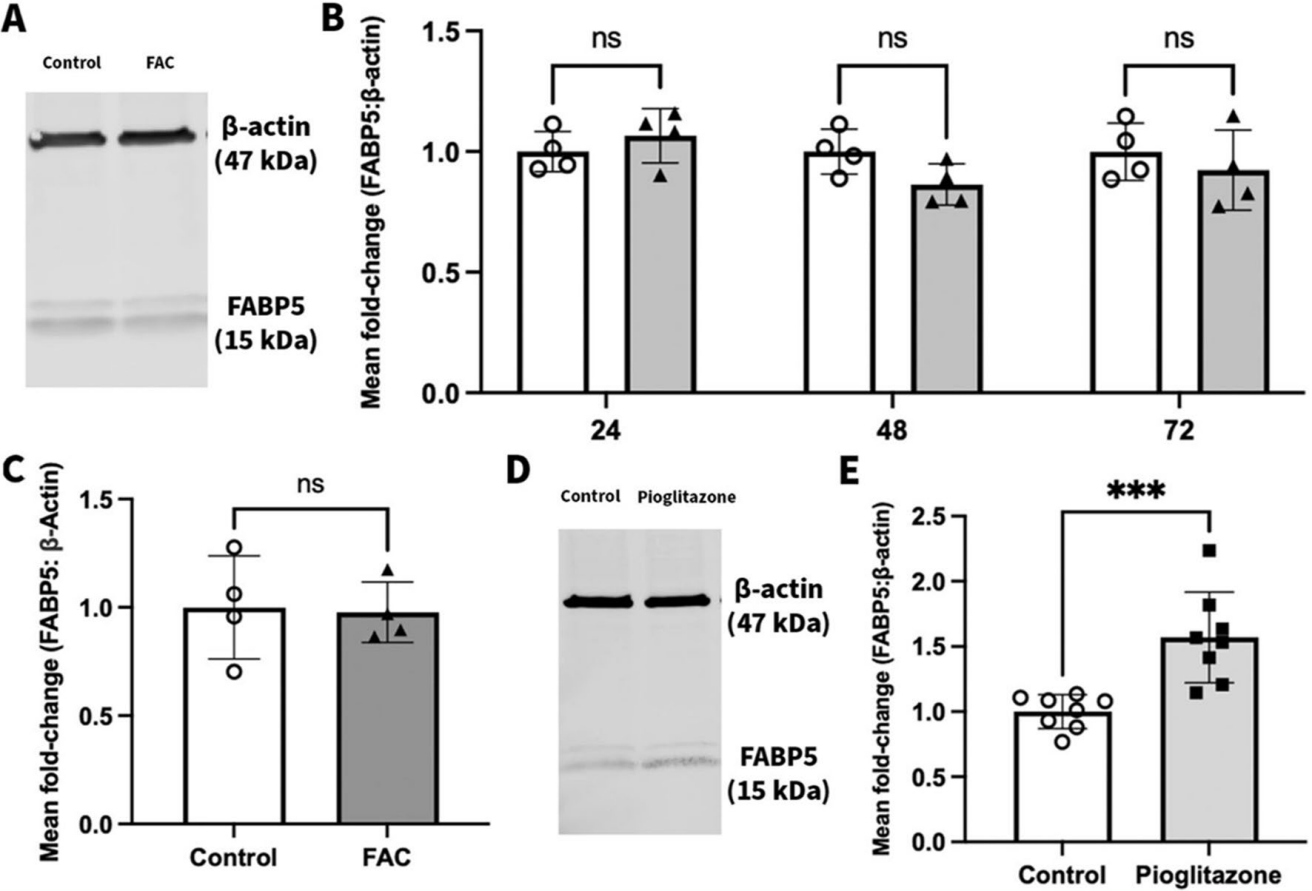
(**A**) A representative western blot demonstrating FABP5 protein abundance in hCMEC/D3 cells treated with vehicle (control) or FAC (500 μM) for 72 h. (**B**) Mean fold-change in FABP5 (15 kDa) protein expression normalised to the housekeeping protein β-actin (47 kDa) in hCMEC/D3 cells treated with vehicle (control; open circles) or FAC (500 μM; filled upward-triangles) for 24 h, 48 h, and 72 h. Data are presented as mean ± SD (*n* = 4, independent cell culture preparations), *p* > 0.05 (non-significant, ns), using a two-way ANOVA test and a post-hoc Šídák's test between control and FAC-treated cells. (**C**) Mean fold-change in FABP5 (15 kDa) protein expression normalised to the housekeeping protein β-actin (47 kDa) in hCMEC/D3 cells treated with vehicle (control; open circles) or FAC (750 μM, 72 h; closed upward-triangles). Data are presented as mean ± SD (*n* = 4, independent cell culture preparations), *p* > 0.05 (non-significant, ns), using a two-tailed Student’s t-test between control and FAC-treated cells. (**D**) A representative western blot demonstrating FABP5 protein abundance in hCMEC/D3 cells treated with vehicle (control) or pioglitazone (25 μM, 72 h). (**E**) Mean fold-change in FABP5 (15 kDa) protein expression normalised to the housekeeping protein β-actin (47 kDa) in hCMEC/D3 cells treated with vehicle (control; open circles) or pioglitazone (25 μM; closed squares) for 72 h. Data are presented as mean ± SD (*n* = 8, independent cell culture preparations), ***p < 0.001, using a two-tailed Student’s t-test between control and pioglitazone-treated cells

To determine whether an overload of iron leads to a change in the protein abundance of FABP5, the hCMEC/D3 cells were treated with FAC at concentrations of 500 µM-750 µM, over multiple treatment periods. A 500 µM concentration of FAC for 24, 48 and 72 h did not affect the abundance of FABP5 protein compared to vehicle treated (control) (Fig. 3A-B). As shown in Fig. 3C, a 750 µM concentration of FAC over 72 h also did not significantly alter FABP5 protein abundance compared to control. The hCMEC/D3 cells treated with pioglitazone (25 μM, 72 h), had an increased protein abundance of FABP5 of 56.9% in comparison to the control cells (Fig. 3D-E). The results obtained from pioglitazone exhibited that FABP5 protein levels could indeed be upregulated via an external stimulus.

### Effect of FAC on the Expression of FABP5 mRNA in hCMEC/D3 Cells

**Fig. 4:**
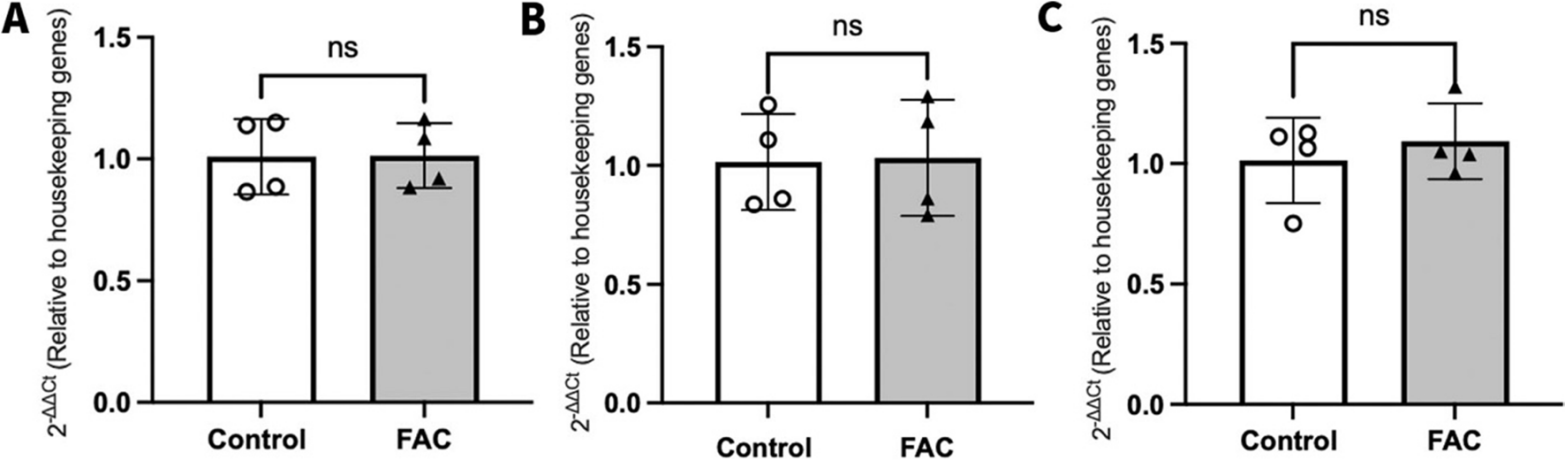
2^−ΔΔCt^ represents the fold-change in FABP5 mRNA transcript levels (normalised to housekeeping genes (GAPDH and β-actin) in RNA isolated from hCMEC/D3 cells treated with vehicle (control; open circles) or FAC (500 μM; closed upward-triangles) for (**A**) 24 h, (**B**) 48 h, or (**C**) 72 h. Data are presented as mean ± SD (*n* = 4, independent cell culture preparations), *p* > 0.05 (non-significant, ns), using a two-tailed Student’s t-test between control and FAC-treated cells

The mRNA expression levels were compared between control and FAC treated hCMEC/D3 cells. Since FAC did not affect the protein abundance of FABP5 at 500 µM and 750 µM concentrations, mRNA was only assessed after a 24–72 h exposure of FAC at a concentration of 500 µM concentration. As shown in Fig. 4, treatments of FAC (500 µM) for 24, 48 and 72 h had no significant impact on FABP5 mRNA expression, in line with a lack of effect of FAC on FABP5 protein abundance.

### Effect of FAC and Pioglitazone on the Uptake of ^3^H-OA and ^14^C-DHA into hCMEC/D3 Cells

**Fig. 5:**
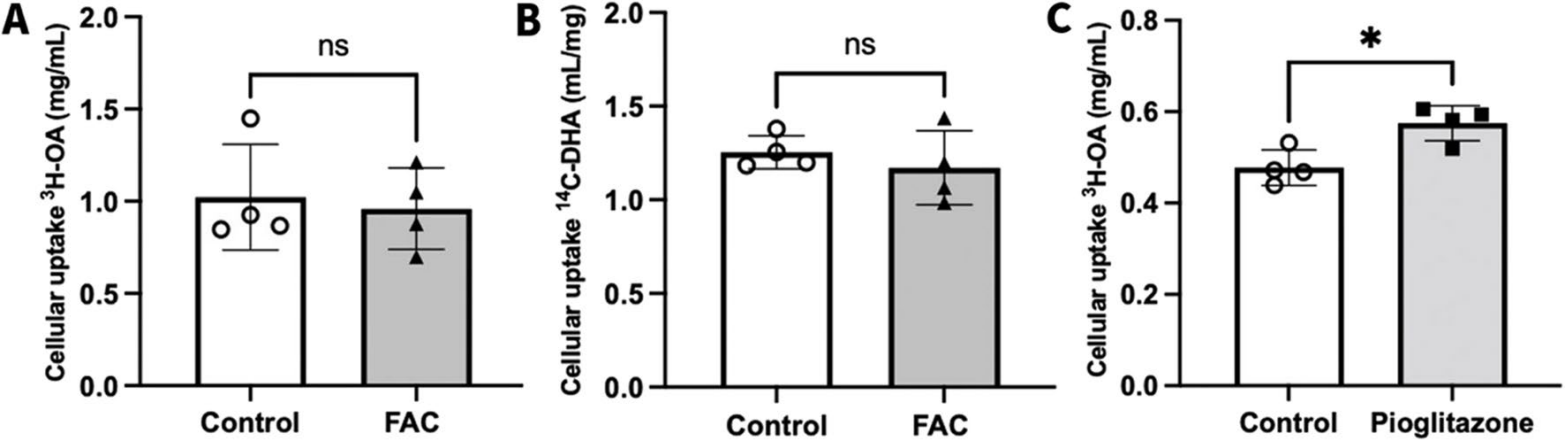
Cellular uptake of (**A**) ^3^H-OA (mL/mg) and (**B**) ^14^C-DHA (mL/mg) in hCMEC/D3 cells, treated with vehicle (control; open circles) or FAC (500 μM; closed upward-triangles) for 72 h. (**C**) Cellular uptake of ^3^H-OA (mL/mg) into hCMEC/D3 cells treated with vehicle (control; open circles) or pioglitazone (25 μM; closed squares) for 72 h. Data are presented as mean ± SD (*n* = 4, independent cell culture preparations), *p < 0.05 and *p* > 0.05 (non-significant, ns), using a two-tailed Student’s t-test between control and treated cells

In line with a lack of effect of FAC on FABP5 abundance, a 72 h treatment with a 500 μM concentration of FAC had no significant effect on the uptake of ^3^H-OA and ^14^C-DHA into hCMEC/D3 cells, as shown in Fig. 5A and B. In contrast, a significant 1.23-fold increase in the uptake of ^3^H-OA was observed in pioglitazone-treated hCMEC/D3 cells relative to control cells (Fig. 5C), in line with the observations that pioglitazone increases the abundance of FABP5. These results demonstrate that FABP5 function can be modulated in hCMEC/D3 cells.

### Effect of FAC on the Abundance of FATP1 Protein in hCMEC/D3 Cells

**Fig. 6:**
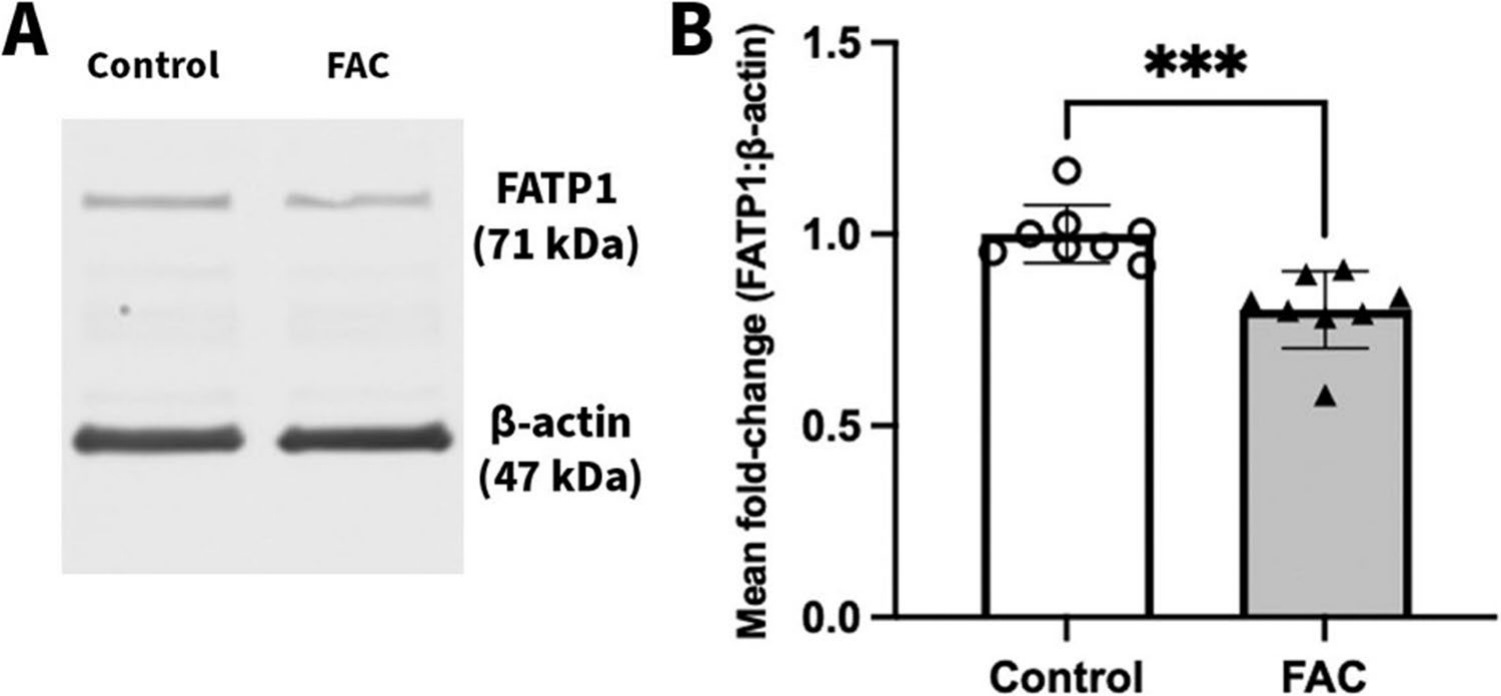
(**A**) A representative western blot demonstrating FATP1 protein abundance in hCMEC/D3 cells treated with vehicle (control) or FAC (500 μM) for 72 h. (**B**) Mean fold-change in FATP1 (71 kDa) protein abundance normalised to the housekeeping protein β-actin (47 kDa) in hCMEC/D3 cells treated with vehicle (control; open circles) or FAC (500 μM; closed upward-triangles) for 72 h. Data are presented as mean ± SD (*n* = 8, independent cell culture preparations), ***p < 0.001, using a two-tailed Student’s t-test between control and FAC-treated cells

To determine whether FAC impacted on the abundance of FATP1, hCMEC/D3 cells were treated with FAC at a concentration of 500 µM concentration for a 72 h treatment period. The abundance of FATP1 was reduced by 19.7% in the FAC-treated cells in comparison to the control cells as shown in Fig. 6.

### Effect of FAC on Efflux of ^3^H-OA and ^14^C-DHA from hCMEC/D3 Cells

**Fig. 7:**
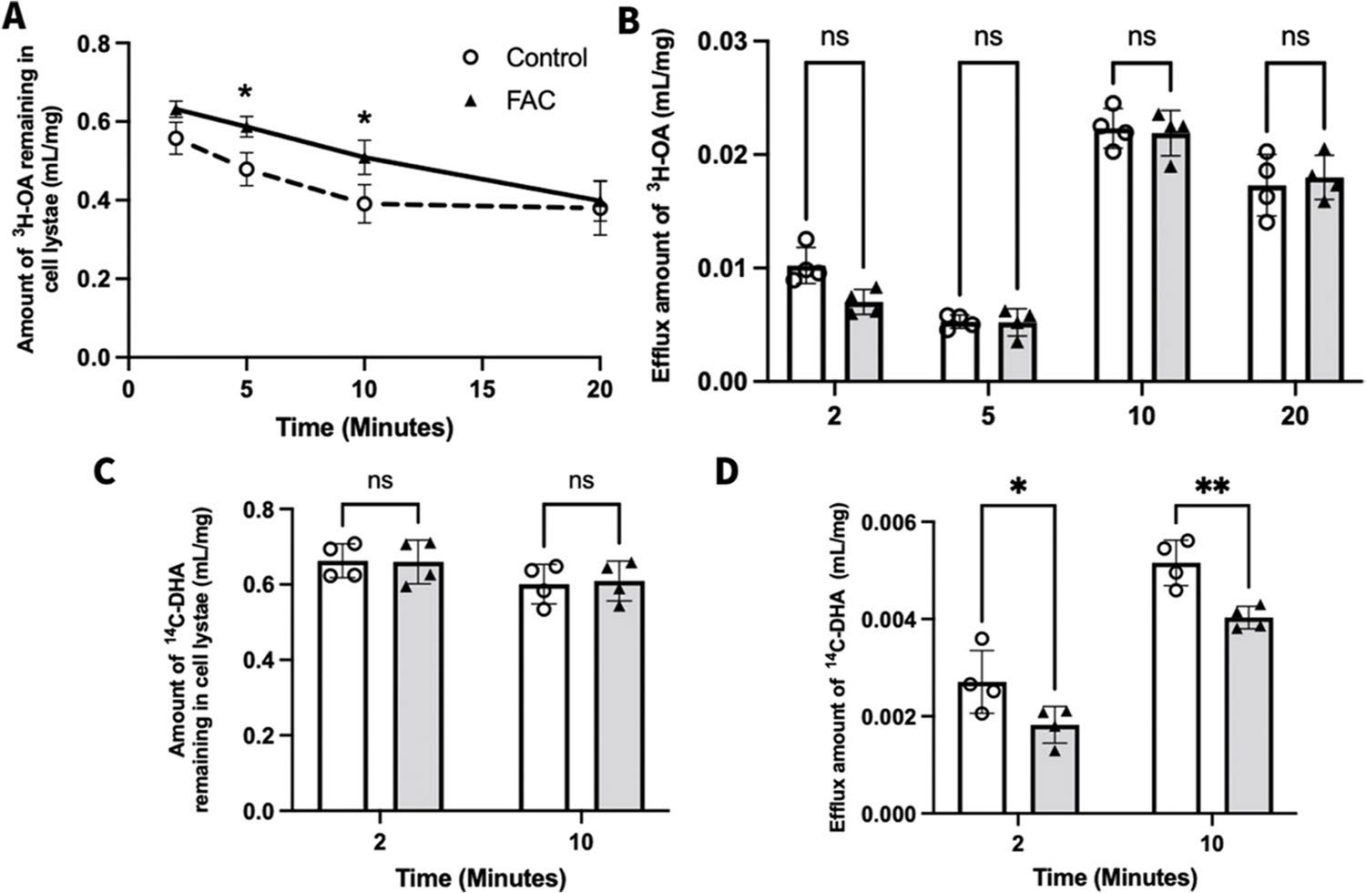
Cellular efflux of ^3^H-OA (mL/mg) and ^14^C-DHA (mL/mg) from hCMEC/D3 cells treated with vehicle (control; open circles) or FAC (500 μM; closed upward-triangles) for 72 h. (**A**) Amount of ^3^H-OA remaining in hCMEC/D3 cell lysate at 2, 5, 10 and 20 min, (**B**) amount of ^3^H-OA detected in the efflux buffer, (**C**) amount of ^14^C- DHA remaining in hCMEC/D3 cell lysate at 2 and 10 min, and (**D**) amount of ^14^C- DHA detected in the efflux buffer. Data are presented as mean ± SD (*n* = 4, independent cell culture preparations), *p < 0.05, **p < 0.01 and p > 0.05 (non-significant, ns), using a two-way ANOVA test and a post-hoc Šídák's test between control and FAC-treated cells

Given that FAC reduced the abundance of FATP1 protein, the effect of FAC on ^3^H-OA and ^14^C-DHA efflux from hCMEC/D3 cells was assessed. The amount of ^3^H-OA remaining in hCMEC/D3 cells rapidly declined for both the control and FAC treated groups until 10 min, with the FAC-treated hCMEC/D3 cells having a higher amount of ^3^H-OA remaining in the cells at 5 and 10 min post efflux in comparison to the amount remaining in the control cells (Fig. 7A). On the other hand, the amount of ^3^H-OA being effluxed out of the control and FAC-treated hCMEC/D3 cells was comparable and had no significant differences at any of the tested time points (Fig. 7B).

When performing the efflux studies with ^14^C-DHA, timepoints of 2 min (lower time point) and 10 min (higher time point) were selected since the amount of ^3^H-OA remaining in the control and FAC-treated hCMEC/D3 cells appeared within the linear phase of the efflux curve (Fig. 7A). In contrast to what was observed for ^3^H-OA, there were no significant differences in the amount of ^14^C-DHA remaining in the hCMEC/D3 cell lysates at the end of the 2 and 10 min efflux experiments (Fig. 7C). On the other hand, at the 2 and 10 min post-efflux timepoint, there was a significantly lower amount of ^14^C-DHA detected in the efflux buffer of FAC-treated hCMEC/D3 cells relative to control hCMEC/D3 cells, as shown in Fig. 7D. These data demonstrate that the FATP1 downregulation induced by FAC was associated with a 22.5% and 30.1% reduction in the efflux of ^3^H-OA at 5 and 10 min, respectively, and a 32.6% and 21.8% reduction in the efflux of ^14^C-DHA at 2 and 10 min, respectively, from the hCMEC/D3 cells.

### Effect of FATP1 siRNA Treatment on the Efflux of ^3^H-OA and ^14^C-DHA from hCMEC/D3 Cells

**Fig. 8:**
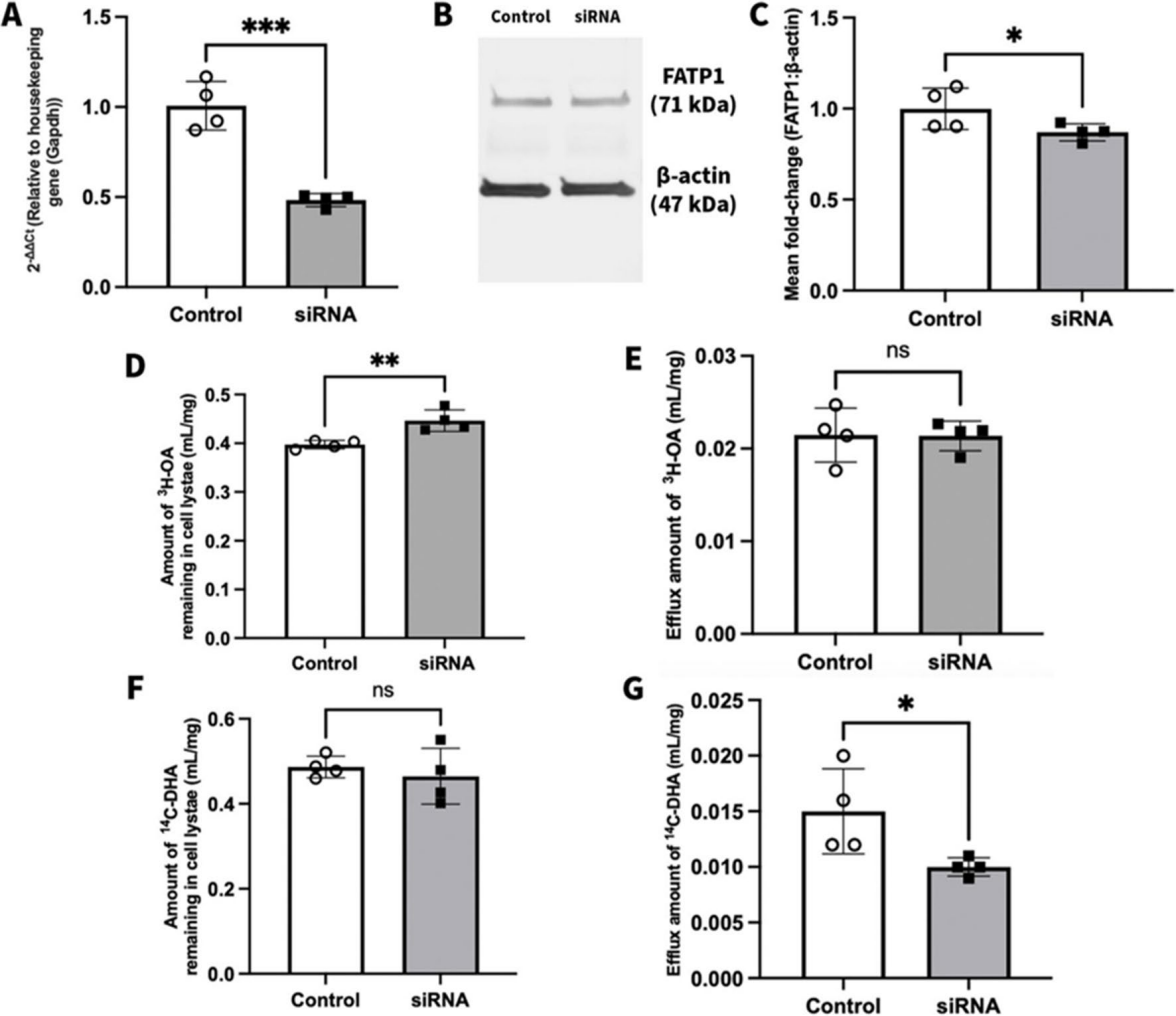
(**A**) 2-ΔΔCt represents the fold-change in FATP1 mRNA transcript levels (normalised to housekeeping gene GAPDH) in mRNA isolated from hCMEC/D3 cells treated with scrambled siRNA (control; open circles) or siRNA against FATP1 (50 nM; closed squares) for 72 h. Data are presented as mean ± SD (*n* = 4, independent cell culture preparations), ***p < 0.001 using a two-tailed Student’s t-test between control and siRNA-treated cells. (**B**) A representative western blot demonstrating FATP1 protein abundance in hCMEC/D3 cells treated with scrambled siRNA (control) or siRNA against FATP1 (50 nM) for 72 h. (**C**) Mean fold-change in FATP1 (71 kDa) protein abundance normalised to the housekeeping protein β-actin (47 kDa) in hCMEC/D3 cells treated with scrambled siRNA (control; open circles) or siRNA against FATP1 (50 nM; closed squares) for 72 h. Data are presented as mean ± SD (*n* = 4, independent cell culture preparations), *p < 0.05, using a one-tailed Student’s t-test between control and siRNA-treated cells. Cellular efflux after 10 min of efflux phase in hCMEC/D3 cells treated with (control; open circles) or siRNA against FATP1 (50 nM; closed squares) for 72 h with (**D**) ^3^H-OA remaining in cell lysate, (**E**) ^3^H-OA in efflux buffer, (**F**) ^14^C-DHA remaining in cell lysate, and (**G**) ^14^C-DHA in efflux buffer. Data are presented as mean ± SD (n = 4, independent cell culture preparations), **p < 0.01, *p < 0.05 and p > 0.05 (non-significant, ns), using a two-tailed Student’s t-test between control and siRNA-treated cells

To confirm the role of FATP1 in fatty acid trafficking, the impact of silencing FATP1 on fatty acid efflux was assessed. A treatment with FATP1 siRNA led to a 51.7% reduction in FATP1 mRNA expression and 13.0% reduction in FATP1 protein abundance in comparison to control, as shown in Fig. 8A-C. The efflux of both ^3^H-OA and ^14^C-DHA from the control and FATP1 siRNA treated hCMEC/D3 cells was then assessed at 10 min post-efflux given that this time point was associated with modified fatty acid trafficking in the studies above (Fig. 7). As shown in Fig. 8D, the amount of ^3^H-OA remaining in hCMEC/D3 cells was 12.4% higher in the siRNA-treated cells in comparison to control cells. However, as was observed following FAC treatment, this was not associated with a reduced amount of ^3^H-OA in the efflux buffer (Fig. 8E). In contrast, but in line with that observed with FAC treatment, FATP1 siRNA treatment led to no significant difference in the amount of ^14^C-DHA remaining in the cell lysates at the end of the 10 min time efflux study, however, there was 33.3% lower amount of ^14^C-DHA detected in the efflux buffer of siRNA treated cells in comparison to control cells (Fig. 8G).

### Effect of FAC on ^3^H-OA Efflux from hCMEC/D3 Cells in the Presence of FATP1 Silencing

**Fig. 9:**
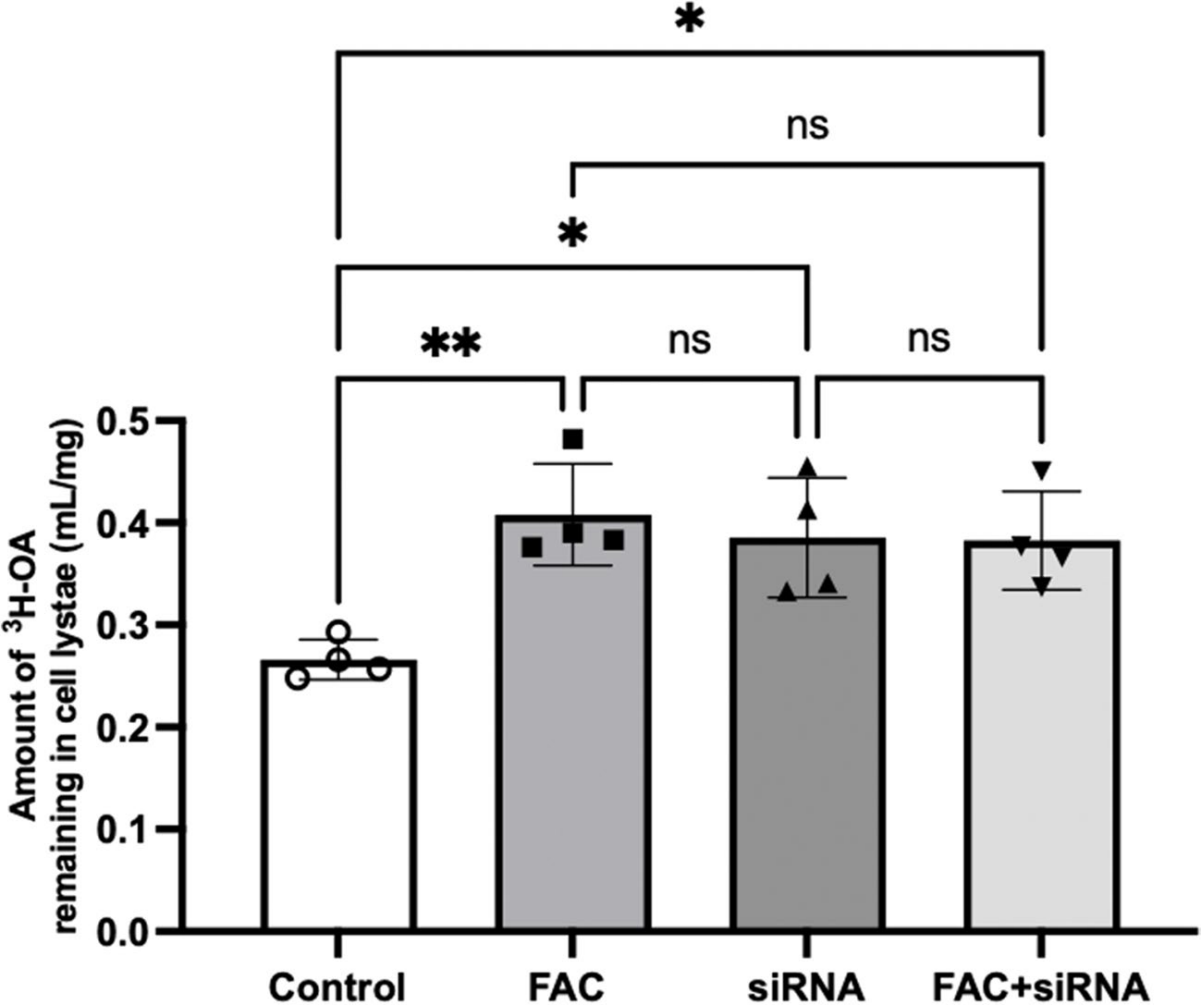
Amount of ^3^H-OA remaining in cell lysate following cellular efflux of ^3^H-OA (mL/mg) in hCMEC/D3 cells treated with vehicle (control; open circles) or FAC (500 μM; closed squares), FATP1 siRNA (50 nM; closed upward-triangles) or combination of FAC and FATP1 siRNA (500 μM and 50 nM; closed downward-triangles) for 72 h. Data are presented as mean ± SD (*n* = 4, independent cell culture preparations), *p < 0.05, **p < 0.01 and *p* > 0.05 (non-significant, ns), using a one-way ANOVA test and a post-hoc Tukey's test where mean of every treatment was compared to all other groups

To assess if the effect of FAC on fatty acid efflux was FATP1-dependent, the efflux of ^3^H-OA was assessed in FAC and FATP1-silenced hCMEC/D3 cells. Figure 9 shows that there was a 1.53-fold increase in ^3^H-OA amount in FAC-treated hCMEC/D3 cell lysates and a 1.45-fold increase in ^3^H-OA amount in FATP1 siRNA-treated hCMEC/D3 cell lysates, relative to control cells. When comparing the effect of FAC in conjunction with FATP1 siRNA treatment, there was a similar 1.44-fold change in ^3^H-OA amount relative to control cells, with no additive effects observed in comparison to the FAC and FATP1 siRNA only groups. However, it should be noted that a complete knockdown of FATP1 with FATP1 siRNA was not achieved (Fig. 8A), which may have been responsible for influencing this effect on efflux.

### Assessing the Mechanism by Which FAC Decreases FATP1 Abundance in hCMEC/D3 Cells

**Fig. 10:**
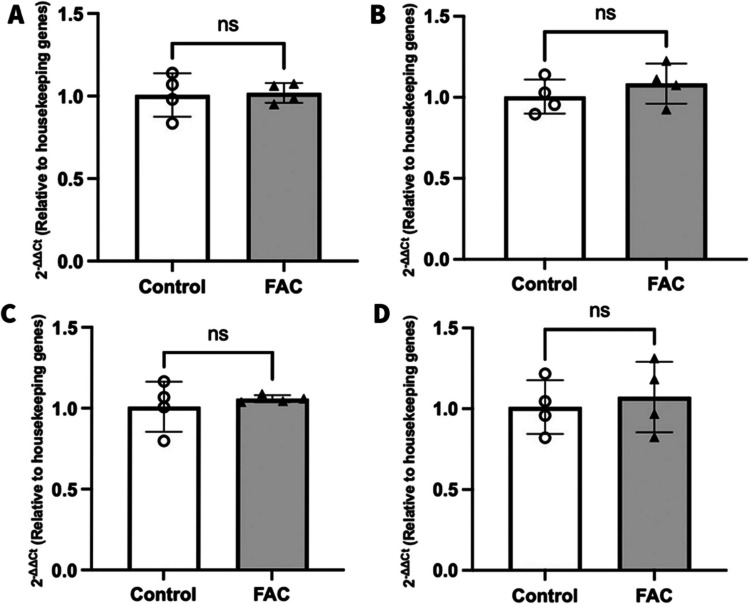
2-ΔΔCt represents the fold-change in FATP1 mRNA transcript levels (normalised to housekeeping genes GAPDH and β-actin) in RNA isolated from hCMEC/D3 cells after vehicle (control; open circles) or FAC (500 μM; closed upward-triangles) treatment for (**A**) 12 h, (**B**) 24 h, (**C**) 48 h, and (**D**) 72 h Data are presented as mean ± SD (*n* = 4, independent cell culture preparations), *p* > 0.05 (non-significant, ns), using a two-tailed Student’s t-test between control and FAC-treated cells

The FATP1 mRNA expression levels were compared between control and FAC-treated hCMEC/D3 cells to assess whether the effects of FAC on FATP1 abundance were transcriptional in nature. As shown in Fig. 10, FAC had no significant impact on FATP1 mRNA expression in comparison to vehicle-treated cells (control) at 12, 24, 48 or 72 h after treatment. This suggests that the impact of FAC on FATP1 protein abundance was not due to transcriptional changes to FATP1 mRNA in hCMEC/D3 cells.

**Fig. 11:**
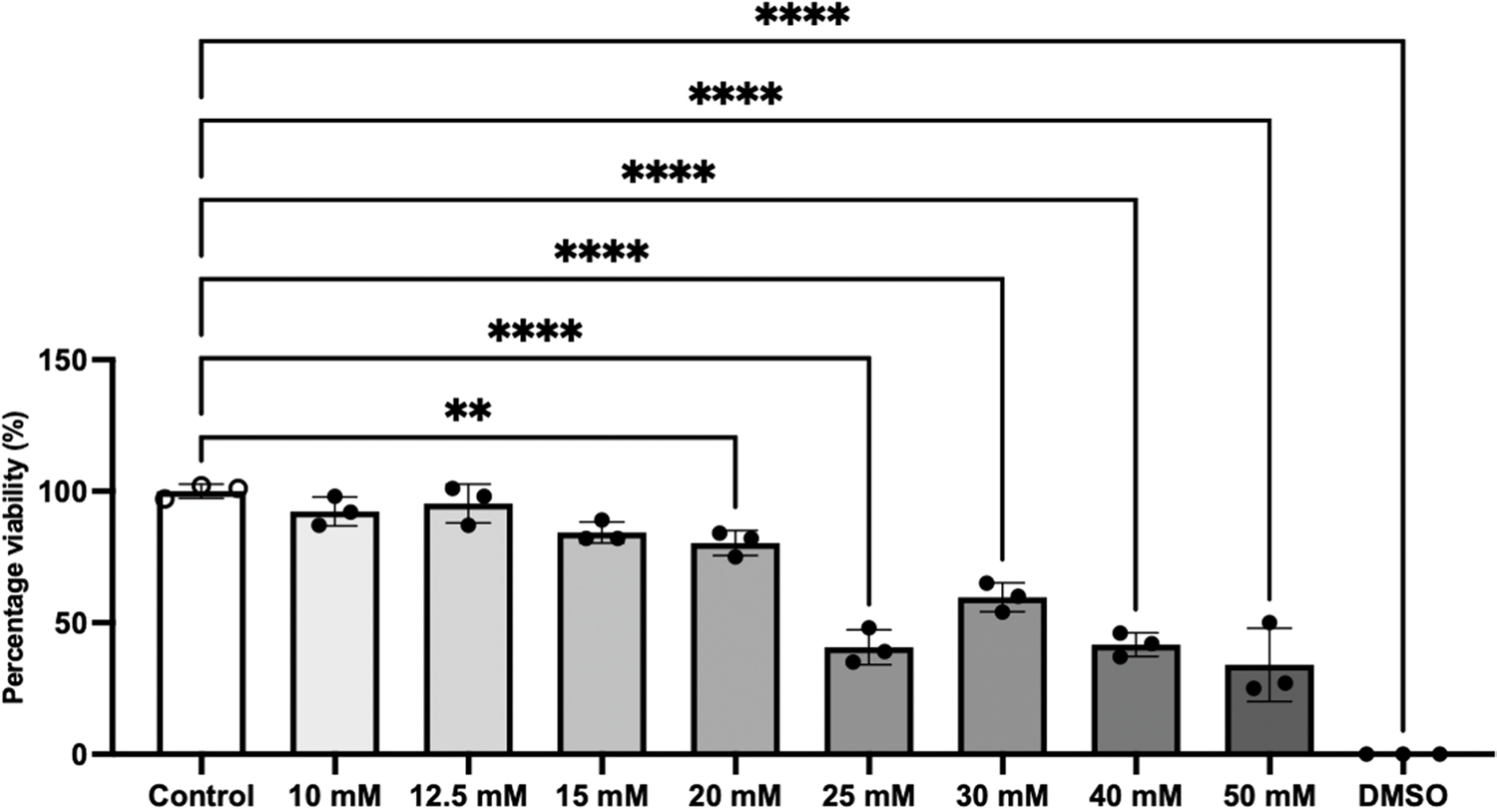
hCMEC/D3 cell viability assay following treatment with vehicle (control), 10 to 50 mM SP concentrations and 10% *(v/v)* DMSO (positive control) over 72 h. Data are presented as mean ± SD (*n* = 3, independent cell culture preparations). **p < 0.01 and ****p < 0.0001, using a one-way ANOVA and a post-hoc Dunnett’s test where the mean of every treatment was compared to the control mean

**Fig. 12:**
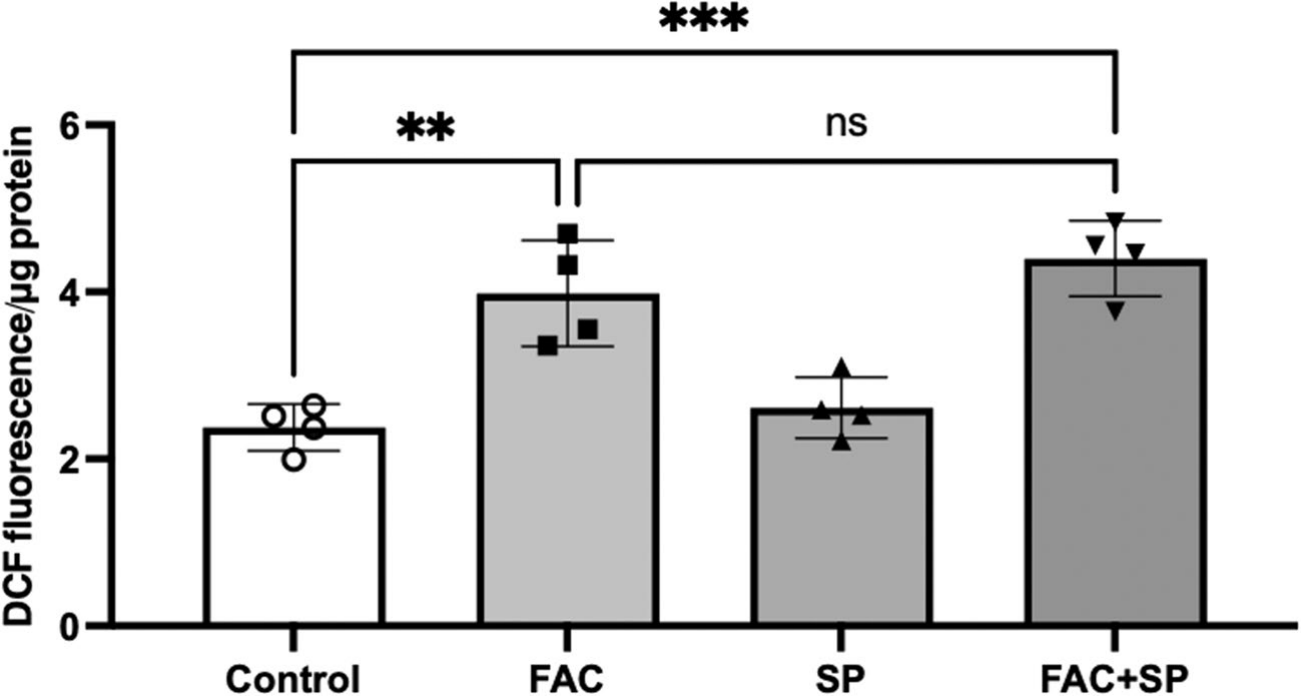
ROS levels in hCMEC/D3 cells following treatment with vehicle (control; open-circles), FAC (500 μM; closed squares), SP (15 mM; closed upward-triangles) and FAC in conjunction with SP (500 μM and 15 mM; closed downward-triangles) for 72 h. Data are presented as mean ± SD (*n* = 4, independent cell culture preparations), ***p < 0.001, **p < 0.01 and *p* > 0.05 (non-significant, ns), using a using a one-way ANOVA and a post-hoc Tukey’s test where the mean of every treatment was compared to all other groups

To assess if the downregulation of FATP1 protein abundance following FAC treatment was due to ROS production, hCMEC/D3 cells were treated with FAC in the presence and absence of the ROS-scavenger, SP. As shown in Fig. 11, concentrations up to 15 mM of SP were found to be tolerable by hCMEC/D3 cells. As shown in Fig. 12, intracellular ROS levels were significantly elevated in the FAC-treated cells, as determined using the intracellular ROS probe DCFH-DA. However, this FAC-mediated increase in ROS was not attenuated by SP at a concentration of 15 mM (Fig. 12), and for this reason, the impact of SP on potentially reversing FAC-mediated changes to FATP1 abundance was not assessed.

**Fig. 13:**
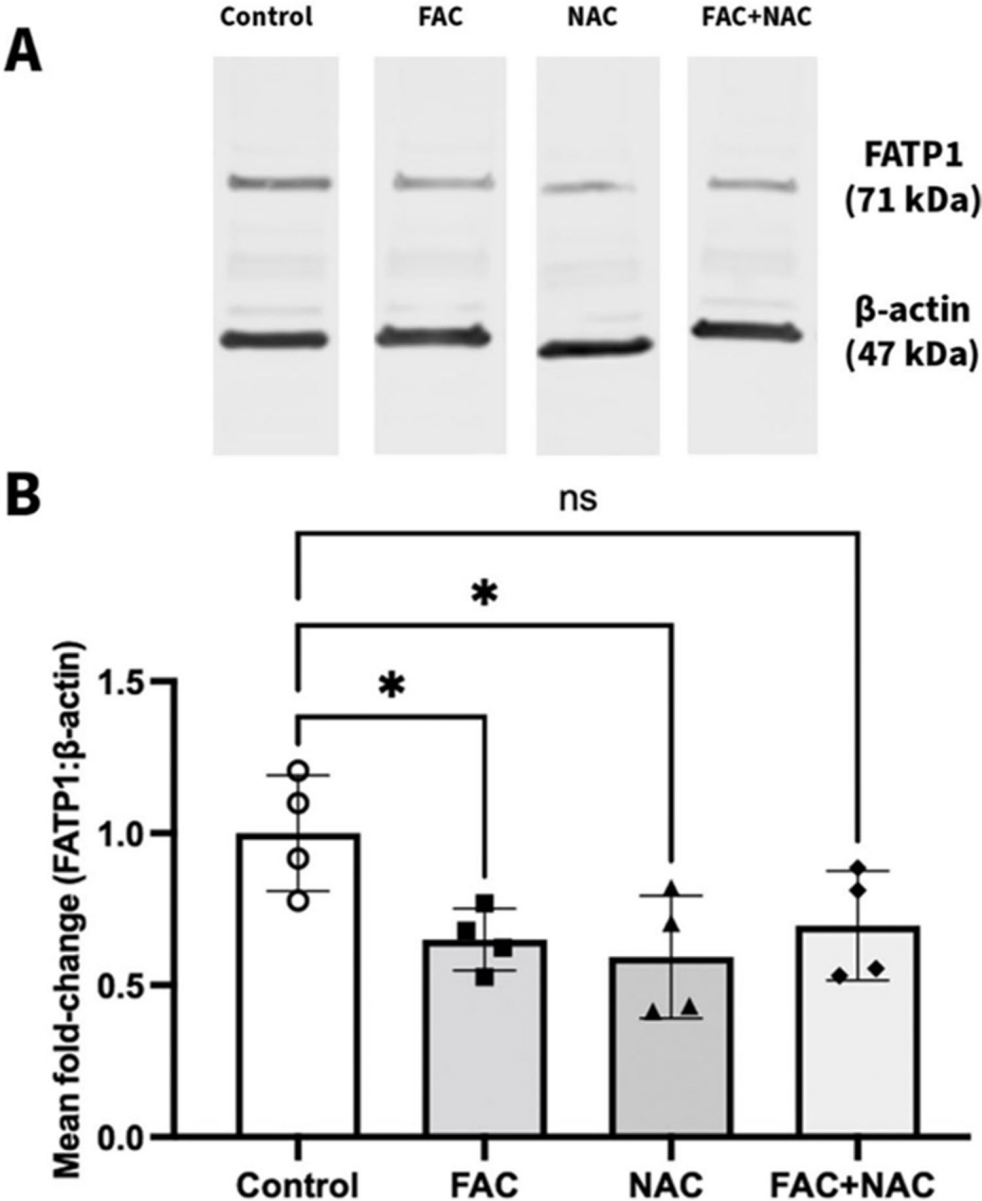
(**A**) A representative western blot demonstrating FATP1 protein in hCMEC/D3 cells treated with vehicle (control), FAC (500 μM), NAC (10 mM) or FAC in combination with NAC (500 μM and 10 mM) for 72 h and (**B**) mean fold-change in FATP1 (71 kDa) protein abundance normalised to the housekeeping protein β-actin (47 kDa) in hCMEC/D3 cells treated with vehicle (control; open-circles), FAC (500 μM; closed squares), NAC (10 mM; closed upward-triangles) or FAC in combination with NAC (500 μM and 10 mM; closed diamonds) for 72 h. Data are presented as mean ± SD (*n* = 4, independent cell culture preparations), *p < 0.05 and *p* > 0.05 (non-significant, ns), using a one-way ANOVA and a post-hoc Dunnett’s test where the mean of every treatment was compared to the control mean

Given that FAC-mediated ROS could not be restored by SP, hCMEC/D3 cells were treated with the more commonly employed ROS scavenger, NAC, at a concentration that had been previously shown to reduce FAC-induced ROS in hCMEC/D3 cells [30]. Figure 13 shows of the abundance of FATP1 in hCMEC/D3 cells after a 72 h treatment with vehicle, FAC, NAC and FAC in combination with NAC. If FAC-induced ROS were responsible for the reduction in FATP1 abundance, it was expected that exposure of NAC would revere the FAC-induced FATP1 reduction. While FAC alone caused a significant down-regulation of FATP1 abundance by 35.0%, NAC alone also caused a similar reduction in FATP1 abundance. Similarly, the combination treatment of FAC and NAC led to a 30.4% reduction in FATP1 abundance, albeit this was not statistically significant. Since NAC alone reduced FATP1 protein abundance, it could not be concluded if the FAC-mediated reduction in FATP1 was indeed ROS-mediated.

## Discussion

There is a growing body of evidence suggesting a link between AD and dysregulation of fatty acid metabolism in the brain and cerebrospinal fluid (CSF). Moreover, apart from the hallmark features of AD, it has also been observed that DHA levels are also reduced in AD brains [1–6]. In plasma, iron is bound to transferrin, however there is a pool of non-transferrin bound iron which has the capacity to generate highly reactive free radicals and is involved in pathological processes [35]. There is a considerable amount of data demonstrating effects of iron on the pathology of AD, but limited understanding of the impact of iron overload on the BBB [23, 36–40]. Previous studies in our laboratory have shown that iron overload led to reduced P-gp protein abundance and BCRP mRNA expression in hCMEC/D3 cells [29, 30], and therefore it was hypothesised that iron may also modify the expression and function of transport processes critical for DHA entry into the brain. Therefore, we determined the effect of iron overload on the expression and function (fatty acid transportation) of FABP5 and FATP1 in hCMEC/D3 cells and investigated mechanisms responsible for driving the observed changes.

The hCMEC/D3 cells used for this study, developed by Weksler *et al*., are a widely used *in vitro* model of the human BBB [41]. The hCMEC/D3 cell line is a valuable model for studying the mechanisms of fatty acid transport, due to its human origin, expression of relevant transporters and functional characteristics [18, 21, 42, 43]. Research has shown that hCMEC/D3 cells can be used to study the uptake and efflux of specific fatty acids, such as DHA [18, 20]. A previous study demonstrated that hCMEC/D3 cells which were treated with 1 mM unlabelled DHA had a 38.6% reduced uptake of ^3^H-OA and when the hCMEC/D3 cells were treated with 1 mM unlabelled OA, the uptake of ^14^C-DHA was reduced by 38.2% [18]. These results suggest that OA and DHA share similar transport processes in hCMEC/D3 cells. Hence, ^3^H-OA was also used, alongside ^14^C-DHA, as a surrogate fatty acid [34] to understand how various treatments impacted on the transportation of fatty acids. The results also exhibit a competitive inhibition of BBB transport of OA and DHA in presence of other PUFAs, hence fatty acid free BSA was utilised for the uptake and efflux studies to mitigate such confounding factors.

An MTT assay was performed on hCMEC/D3 cells to identify the non-toxic concentrations of FAC, and it was identified that the highest tolerable cocentration was 1500 μM over a 72 h treatment, which formed the basis of choosing the concentrations for all subsequent experiments in this study. The decrease in cell viability between 1500 µM and 2000 µM concentrations of FAC could be due to a concentration-dependent effect on cell toxicity. At lower concentrations, the hCMEC/D3 cells appear to be able to manage the FAC-induced stress, but beyond a certain concentration, FAC overwhelms cellular defence mechanisms, leading to a sharp decline in viability. The 2000 µM concentration of FAC clearly reached a toxicity threshold where it becomes significantly toxic to the cells. Multiple concentrations of FAC ranging between 100 μM to 750 μM over multiple treatment periods ranging between 24 to 72 h were tested for mRNA and protein abundance studies however, we report only the results from studies using 500–750 μM of FAC, as results from these studies were similar to those observed with lower concentrations of FAC. Initially, a concentration of 500 µM FAC was chosen based on previous studies in U251 and SH-SY5Y cells to mimic iron overload [44]. The higher FAC concentration of 750 μM was employed as the concentration of iron reported by Lovell *et al.* in AD brain was 38.8 μg/g, which translates ~ 695 μM [45] and a review article reported approximate 700 μM concentration of iron in brain parenchyma of individuals with AD [46]. Given the lack of change of FABP5 as a result of FAC treatment at these concentrations, it was critical to confirm that FABP5 could be regulated via an external stimulus in hCMEC/D3 cells. For this reason, pioglitazone (a known PPARγ agonist) was used to treat the hCMEC/D3 cells [21]. The pioglitazone treatment in this study led to a significant increase in FABP5 protein abundance and uptake function (uptake of ^3^H-OA) in hCMEC/D3 cells, compared to control cells. ^3^H-OA was used as surrogate for DHA, since the two fatty acids are known to share similar transport processes [34]. The findings in this study with pioglitazone are in line with a previous study conducted on hCMEC/D3 cells [21], suggesting that indeed FABP5 can be regulated via an external stimulus and that the lack of effect of FAC on FABP5 was not a result of FABP5 being non-responsive to external stimuli.

Several other fatty acid transporters including fatty acid translocase (CD36), major facilitator superfamily domain-containing protein 2a (Mfsd2a) and FATP4 have also been suggested to facilitate the transport of fatty acids. However, in studies conducted in HBMECs and mouse brain homogenates, CD36 has been identified to have a lower expression in comparison to FATP1 and FATP4 [13]. Moreover, CD36^−/−^ mice did not present with a significant difference in DHA concentrations in the different brain regions in comparison to wild-type control mice [47], suggesting that CD36 may not be essential to maintain brain DHA levels. On the other hand, Mfsd2a is well-known in the literature to play an important role in the uptake of esterified DHA [10, 48, 49], whereas non-esterified DHA is the major pool supplying DHA to the brain [10, 49]. Hence, Mfsd2a was not explored as a part of this study. The current literature indicates that FATP1 appears to be expressed more on the brain-facing membrane of the brain microvasculature and furthermore that FATP1 is involved in the efflux of DHA [18, 50]. Hence to further understand whether iron may impact on overall brain uptake of fatty acids, the impact on FAC on FATP1 became the focus of this study.

Our studies demonstrated that FAC indeed downregulated the protein abundance of FATP1 (Fig. 6). When assessing the variability associated with the FATP1 abundance in vehicle treated cells, we identified that the level of variability was 4.4%. Therefore, we are confident that the approximate 20% reduction in FATP1 abundance mediated by FAC is outside the nominal variability of the method employed. Interestingly, FAC had not decreased the uptake of ^3^H-OA and ^14^C-DHA from hCMEC/D3 cells suggesting FATP1 is not involved in uptake of fatty acids because if it were, then FAC would have been expected to reduce the hCMEC/D3 uptake of fatty acids. Given the suggestions that FATP1 is involved in mediating efflux of DHA from hCMEC/D3 cells [20], the efflux of ^3^H-OA and ^14^C-DHA from hCMEC/D3 cells following FAC treatment was assessed. It was observed that the FAC treated cells, in comparison to control cells, had a significantly higher amount of ^3^H-OA remaining in the cell lysates at the end of the 5 and 10 min time points. This suggested that there was lesser efflux of ^3^H-OA from the FAC treated cells, even though there was not a significant difference observed in the amount of ^3^H-OA appearing in the efflux buffer. Moreover, in studies performed using ^14^C-DHA, there was a significantly lower amount of ^14^C-DHA being released into the efflux buffer from the FAC-treated cells in comparison to the control cells, confirming reduced efflux of ^14^C-DHA with FAC treatment. However, interestingly unlike for ^3^H-OA, there was no significant difference in the amount of ^14^C-DHA remaining in the cell lysates with FAC treatment. The exact reason for the discrepancy between these measurements remains unknown. It is possible that the affinity of each substrate to elements of the *in vitro* system differ and, therefore, the results were obscured. The equilibrium dissociation constants (*K*_d_) of OA and DHA for BSA, which is in the cell media and buffers used in performing the efflux studies, have been reported to be 6.6 nM and 29 nM at 38°C, respectively [51, 52]. Moreover, there is evidence in the literature suggesting that OA and DHA have differential susceptibility for peroxidation due to the difference in unsaturation index [53]. This difference in binding affinities for BSA and susceptibility for peroxidation, alongside a potential difference in intracellular binding and adsorption to the cell culture plasticware, could be potential explanations of the complementary yet different observations for ^3^H-OA and ^14^C-DHA in the efflux studies. Nonetheless, these functional studies suggest that FAC impacts on the function of proteins involved in fatty acid efflux *in vitro*.

The findings from this study show that FAC induced iron overload led to a reduction in the abundance of FATP1 protein and a reduced efflux of ^3^H-OA and ^14^C-DHA from hCMEC/D3 cells. These findings support the notion that FATP1 is involved in the efflux of fatty acids [18, 20]. To further investigate the importance of FATP1 in the trafficking of fatty acids out of hCMEC/D3 cells, FATP1 siRNA was used. However, it should be noted that the siRNA treatment had a lower effect on FATP1 protein abundance, in comparison to the effect that it had on mRNA expression Nonetheless, it was observed that at the end of a 10 min efflux period, the efflux of ^3^H-OA and ^14^C-DHA was significantly lower in the FATP1 siRNA treated cells in comparison to control cells. This study further establishes that FATP1 plays a crucial role in the efflux of ^3^H-OA and ^14^C-DHA from hCMEC/D3 cells. Our studies also show that the combination of FATP1 siRNA and FAC were not additive in terms of influencing OA efflux, suggesting that the changes in efflux function induced by FAC treatment were most likely mediated by FATP1 downregulation. However, since a complete knockdown of the FATP1 protein was not achieved following siRNA treatment and no additive effects on efflux were observed following combination treatment of FAC and siRNA, we cannot conclude whether the effects of FAC on efflux of fatty acids was only due to FATP1 or whether there is involvement of other trafficking proteins partly contributing to the effects of FAC. Since FATP1 is known to be preferentially expressed in the abluminal membrane of brain endothelial cells [18] and iron overload is observed in AD [23, 24], these findings provide an insight on how iron overload can be a contributing factor to the reduction in brain DHA levels observed in AD stemming from reduced the efflux of DHA from the brain endothelial cells into the brain.

Having established a potential role of FATP1 in OA and DHA efflux from hCMEC/D3 cells, it was critical to evaluate the mechanism by which FAC was reducing the abundance of FATP1 protein. We demonstrated that FAC did not impact on the mRNA levels of FATP1 in hCMEC/D3 cells, suggesting the impact of FAC on the expression of FATP1 may not be transcriptionally driven. Iron is a well-known inducer of ROS [28, 54] and in previous *in vitro* studies with hCMEC/D3 cells, FAC at the concentration employed in this study has been shown to increase intracellular ROS [29]. Increased ROS levels have been associated with changes in the permeability of the BBB and redistribution or alterations in the expression of critical tight junction proteins such as claudin-5 and occludin [55, 56]. Hence, to understand if the down-regulation of FATP1 protein following FAC treatment was mediated by ROS, the hCMEC/D3 cells were treated with FAC in the presence and absence of SP, a known ROS scavenger [33]. Despite testing the highest tolerable concentrations of SP, this intervention was unable to counteract the ROS produced due to FAC. Therefore, further experiments were not conducted using SP. Excess iron via the Fenton and Haber–Weiss reactions leads to the generation of different ROS, including hydroxyl/lipid peroxyl radicals, superoxide radicals, and hydrogen/lipid peroxides [57]. SP is known to act as a potent intracellular scavenger for hydrogen peroxide [33], and it was considered that the effects of FAC could be mediated by alternative ROS which could not be attenuated by SP.

For this reason, another commonly used cytosolic antioxidant/ROS scavenger NAC was then trialled [28, 29, 58]. It was previously shown that ROS induced by FAC *in vitro* could be attenuated by NAC [29]**.** In this study, it was demonstrated that NAC alone elicited a significant reduction in FATP1 abundance. Hence, this experiment could not conclude that the impact of FAC on the abundance of FATP1 was fully recovered by the presence of an antioxidant, since NAC itself caused a down-regulation of FATP1 protein, due to unknown mechanisms. However, these are the first studies to demonstrate that ROS alone could be involved in regulating FATP1 abundance given that NAC alone was able to modulate FATP1 levels, an area of research requiring further investigation. Further studies are required to elucidate the cellular mechanisms by which FAC downregulates FATP1, perhaps via exploring the impact of FAC on post-transcriptional regulation of FATP1 since this study as already established that FAC had no impact on FATP1 mRNA levels.

The findings from this study strengthen the current literature regarding the importance of FATP1 in the trafficking of OA and DHA from human brain capillary endothelial cells. This study indicates that the abundance and efflux activity of FATP1 protein is reduced due to the impact of iron overload caused by FAC in human brain capillary endothelial cells, with FAC having no effect on FABP5 abundance and function. These findings can be helpful in understanding the pathological effects observed at the BBB in AD. Since the current study was performed using a simple *in vitro* model, the findings represent an acute setting which may not the replicate the complexities of an *in vivo* disease state. Therefore, further studies assessing the impact of FAC on the BBB expression and function of FATP1 could be conducted in healthy mice, mice with iron overload and AD mouse models to evaluate the disease relevance of these findings *in vivo.*

## Conclusions

In conclusion, the data from the present study are the first to demonstrate that increasing intracellular iron levels using FAC led to downregulation of FATP1 at the protein level, but not the mRNA level, in a human model of the BBB. Furthermore, upon performing functional studies it was observed that FAC had no impact on the uptake of ^3^H-OA and ^14^C-DHA, but significantly reduced the efflux of ^3^H-OA and ^14^C-DHA *in vitro*. While further studies are required to elucidate the molecular mechanisms underlying the FAC-induced downregulation of FATP1 and *in vivo* studies are required to assess the translatability of these findings, this study provides insight into a role of iron in regulating FATP1 abundance and function at the BBB, which may have implications on the transport of fatty acids to the brain.

## Data Availability

The data that support the findings of this study are available from the corresponding author upon reasonable request.

## Abbreviations

AD: Alzheimer’s disease
BBB: Blood–brain barrier
BCRP: Breast cancer resistance protein
CD36: Fatty acid translocase
CNS: Central nervous system
CSF: Cerebrospinal fluid
DCFH-DA: 2′,7′-Dichlorofluorescin diacetate
DHA: Docosahexaenoic acid
FABP5: Fatty acid binding protein 5
FAC: Ferric ammonium citrate
FATP1: Fatty acid transport protein 1
FATP4: Fatty acid transport protein 4
hCMEC/D3: Immortalised human cerebral microvascular endothelial cell line
Mfsd2a: Major facilitator superfamily domain-containing protein 2a
NAC: N-Acetyl-L-cysteine
OA: Oleic acid
P-gp: P-glycoprotein
PPARs: Peroxisome proliferator-activated receptors
PUFA: Polyunsaturated fatty acid
ROS: Reactive oxygen species
SP: Sodium pyruvate
